# Spotlight influenza: Influenza surveillance before and after the introduction of point-of-care testing in Denmark, season 2014/15 to 2018/19

**DOI:** 10.2807/1560-7917.ES.2021.26.37.2000724

**Published:** 2021-09-16

**Authors:** Guido Benedetti, Tyra Grove Krause, Uffe Vest Schneider, Jan Gorm Lisby, Marianne Voldstedlund, Didi Bang, Ramona Trebbien, Hanne-Dorthe Emborg

**Affiliations:** 1 Department of Infectious Disease Epidemiology and Prevention, Statens Serum Institut, Copenhagen, Denmark; 2 European Programme for Intervention Epidemiology Training (EPIET), European Centre for Disease Prevention and Control, (ECDC), Stockholm, Sweden; 3 Department of Clinical Microbiology, Rigshospitalet, University of Copenhagen, Copenhagen, Denmark; 4 Department of Clinical Microbiology, Copenhagen University Hospital, Amager and Hvidovre, Copenhagen, Denmark; 5 Department of Data Integration and Analysis, Statens Serum Institut, Copenhagen, Denmark; 6 Department of Virus and Microbiological Special Diagnostics, Statens Serum Institut, Copenhagen, Denmark

**Keywords:** point-of-care testing, influenza, surveillance, Denmark

## Abstract

**Background:**

In Denmark, influenza surveillance is ensured by data capturing from existing population-based registers. Since 2017, point-of-care (POC) testing has been implemented outside the regional clinical microbiology departments (CMD).

**Aim:**

We aimed to assess influenza laboratory results in view of the introduction of POC testing.

**Methods:**

We retrospectively observed routine surveillance data on national influenza tests before and after the introduction of POC testing as available in the Danish Microbiological Database. Also, we conducted a questionnaire study among Danish CMD about influenza diagnostics.

**Results:**

Between the seasons 2014/15 and 2018/19, 199,744 influenza tests were performed in Denmark of which 44,161 were positive (22%). After the introduction of POC testing, the overall percentage of positive influenza tests per season did not decrease. The seasonal influenza test incidence was higher in all observed age groups. The number of operating testing platforms placed outside a CMD and with an instrument analytical time ≤ 3 h increased after 2017. Regionally, the number of tests registered as POC in the Danish Microbiological Database and the number of tests performed with an instrument analytical time ≤ 3 h or outside a CMD partially differed. Where comparable (71% of tests), the relative proportion of POC tests out of all tests increased from season 2017/18 to 2018/19. In both seasons, the percentage of positive POC tests resulted slightly lower than for non-POC tests.

**Conclusion:**

POC testing integrated seamlessly into national influenza surveillance. We propose the use of POC results in the routine surveillance of seasonal influenza.

## Introduction

Rapid diagnostic testing of patients performed outside a central laboratory has been termed point-of-care (POC) testing or ‘near patient testing’. POC testing means processing patient material in different geographical settings outside the clinical microbiological department e.g. in the hospital emergency room or at general practice [1,2]. Several commercially available POC platforms have recently been introduced to the market and are increasingly being used in the diagnosis of respiratory tract infections in Europe [3,4]. Studies have shown its potential role in reducing unnecessary antibiotic treatments, ensuring timely antiviral treatment, improving the utilisation of isolation facilities and reducing associated hospitalisation costs [4,5].

Influenza is a worldwide threat to populations’ health, health systems and economies. In Europe, influenza is still a major contributor to mortality, especially among elderly people [6,7]. Access to timely influenza surveillance data is crucial for public health preparedness and response, including resource allocation for seasonal influenza and in a pandemic scenario [8].

In Denmark, the surveillance of influenza is ensured by data capturing from existing population-based registers [9]. Weekly, individual-level information on all diagnostic influenza test results is retrieved from the Danish Microbiology Database (MiBa) [10,11] and linked to hospitalisation and intensive care admissions data from the national patient register in order to assess the influenza burden and severity. Also, the laboratory results of influenza tests in MiBa are linked with data from the Danish vaccination registry to estimate vaccination effectiveness [12].

Since 2017, POC testing has been implemented in Denmark outside the regional clinical microbiology departments (CMD), in hospital wards and outpatient settings using a number of different platforms and setups, according to the national guidelines by the Danish Society for Clinical Microbiology [13]. Standard operating procedures are applied, and local POC committees work in collaboration with the CMD to ensure best practices, including the timely and complete registration of all influenza POC tests in MiBa. The Danish Society of Clinical Microbiology defines POC testing as ‘infectious disease diagnostic procedures performed outside the geography of the Clinical Microbiology Laboratory’. The CMD supply information in MiBa as to whether a test is performed as POC. However, this practice is not monitored and the turnaround time of tests and the placement of platforms is not documented in MiBa.

During the 2017/18 and 2018/19 influenza seasons and after POC testing was introduced, the number of influenza tests and the number of influenza-related hospitalisations registered by surveillance increased substantially in Denmark [14,15]. However, influenza laboratory results have not yet been assessed in view of the introduction of POC testing in Danish healthcare facilities. Therefore, we described the trends in influenza testing by season, region and age group according to POC testing availability. Further, we validated the information on POC testing as provided in MiBa with additional information collected from CMD in Denmark from 2014 to 2019.

## Methods

### Study design

This was a retrospective observational study of routinely collected surveillance data on all national influenza tests before and after the introduction of POC testing in Denmark in the period 2014 to 2019. Furthermore, we performed a questionnaire study among all Danish CMD on influenza diagnostics. 

### Data sources

#### MiBa data

We extracted data on all influenza test results (primary and hospital care) from 2014 (week 1) to 2019 (up to week 20). Individual level data were available for every test, including personal identifiers, sex, age, date of testing, test result (positive/negative), influenza type (A, B, C) and the CMD of reference. Data on whether the test was classified as POC (yes/no) were either retrieved from information about the analysis performed or from a commentary field by searching for pre-identified keywords, i.e. ‘POC’, ‘POCT’, ‘Point’ and ‘Care’.

#### Questionnaire data

Information on the used platforms for influenza testing (brand), the related instrument analytical time (i.e. the time to process a sample according to the manufacturer’s specifications), the number of performed tests per season and the number of locations where testing platforms were running (inside or outside the CMD) was collected from all 11 CMD in Denmark through a semistructured questionnaire covering the period from season 2014/15 to season 2018/19. The questionnaire was developed at Statens Serum Institut in collaboration with the CMD at Copenhagen University Hospital, Amager and Hvidovre and circulated to CMD in June 2019.

#### Statistics Denmark

From Statistics Denmark, we extracted publicly available information on age-specific population size [16].

### Data analysis

The MiBa individual test data were aggregated by week and season and summarised as the number of performed influenza tests, the number of positive influenza tests and percentage positive. Data were displayed by region and age group (categorised as < 5, 5–18, 19–65 and > 65 years). An influenza season was defined as a series of 33 consecutive weeks from week 40 in one year to week 20 in the following year. We defined seasons 2014/15, 2015/16 and 2016/17 as ‘non-POC seasons’ and seasons 2017/18 and 2018/19 as ‘POC seasons’, despite the fact that one CMD piloted POC testing in season 2016/17. Duplicate observations were excluded from analysis, i.e. notifications with the same unique identifier, date of sampling, reporting CMD, result and type of tests (POC or non-POC). In contrast, the number of non-POC tests that had a correspondent POC test (same unique identifier, date of sampling and reporting CMD) was counted per season and included in the analysis. 

The seasonal influenza test incidence was measured as the seasonal number of tests performed per age group over the available age-specific population on 1 January of the relevant season. The mean (with standard deviation (SD)) seasonal percentage of positive tests at regional level before and after the introduction of POC testing was compared by unpaired t-test. Data from the reference laboratory at Statens Serum Institut were excluded from the analysis as it primarily functions as a reference laboratory and analyses samples such as those already tested by regional CMD.

The CMD questionnaire summarised the seasonal number of tests performed per region, instrument analytical time (≤ 3 h or > 3 h) and placement of the testing platform (inside or outside the CMD). An instrument analytical time ≤ 3 h and placement of the testing platform outside of the CMD were considered as proxies of POC tests. We compared the number of POC tests registered in MiBa (individual tests data) against the number of tests with an instrument analytical time ≤ 3 h (CMD questionnaire aggregated data) in seasons 2017/18 and 2018/19 per region. Whenever the number of POC tests registered in MiBa and the number of tests with an instrument analytical time ≤ 3 h were less than 5% different, data were cumulated and displayed in a weekly time series.

As the objective of this analysis was not to assess the performance of different CMD in influenza surveillance, data were aggregated by region in Denmark. Data were imported, managed and analysed in R RStudio Version 1.1.456 [17]. Graphs were created with an Excel electronic worksheet.

### Ethical statement

No ethical approval was required for this register -based study.

## Results

### Danish Microbiology Database individual tests data

**Figure 1:**
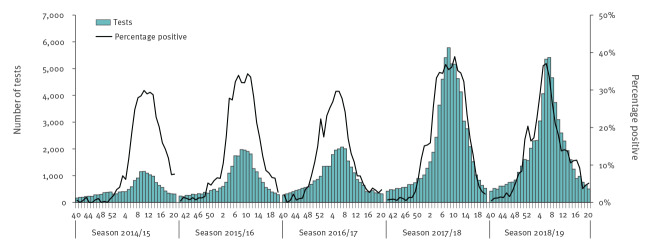
Seasonal weekly number of influenza tests and percentage positive, Denmark, 2014–2019 (n = 199,744)

Source: Danish Microbiological Database.

Overall, 199,744 influenza tests were performed in Denmark from season 2014/15 to season 2018/19, of which 44,161 were positive (22%). The CMD in the Capital Region of Denmark alone accounted for 98,405 tests (49% of the total) with 20,383 positive (21%). Across the country, influenza seasons 2017/18 and 2018/19 alone accounted for 127,347 tests (64% of the total) with 30,686 positive (24%). The seasonal number of influenza tests, positive tests and percentage positive are shown in Figure 1, and by region and age group in Table 1. 

**Table 1:** Seasonal number of influenza tests, positive tests and percentage positive by region and age group, Denmark, 2014–2019 (n = 199,744)

	Season 2014/15			Season 2015/16			Season 2016/17			Season 2017/18			Season 2018/19		
	Pos	Tests	% pos	Pos	Tests	% pos	Pos	Tests	% pos	Pos	Tests	% pos	Pos	Tests	% pos
Denmark	2,842	16,692	17	5,695	26,004	22	4,938	29,701	17	18,244	66,488	27	12,442	60,859	20
R1	1,171	8,067	15	2,615	12,514	21	2,146	14,721	15	8,642	33,560	26	5,809	29,543	20
R2	215	775	28	282	1,114	25	381	1,356	28	1,087	3,247	33	600	2,295	26
R3	377	2,532	15	1,150	4,712	24	578	3,620	16	2,390	8,488	28	1,869	8,170	23
R4	915	4,722	19	1,259	6,033	21	1,164	7,036	17	3,980	14,861	27	2,533	14,034	18
R5	164	596	28	389	1,631	24	669	2,968	23	2,145	6,332	34	1,631	6,817	24
0–4 years	284	3,765	8	725	4,897	15	271	4,869	6	1,125	8,635	13	1,712	10,369	17
5–18 years	208	1,310	16	786	2,442	32	441	2,114	21	1,633	4,610	35	1,230	4,121	30
19–65 years	1,446	6,931	21	2,810	11,171	25	1,921	11,948	16	8,323	27,573	30	5,994	25,581	23
> 65 years	904	4,686	19	1,374	7,494	18	2,305	10,770	21	7,163	25,670	28	3,506	20,788	17

% pos: percentage of positive tests; Pos: positive tests; R1: Capital Region of Denmark; R2: North Denmark Region; R3: Central Denmark Region; R4: Region of Southern Denmark; R5: Region Zealand.

Source: Danish Microbiological Database.

**Figure 2:**
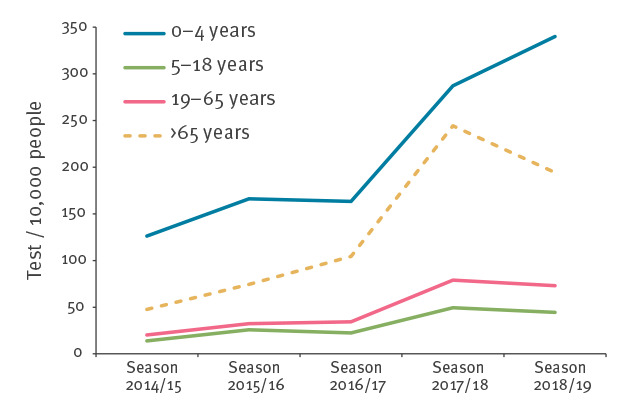
Seasonal influenza test incidence by age group, Denmark, 2014–2019 (n = 199,744)

Source: Danish Microbiological Database.

During season 2016/17, one CMD registered 207 POC tests in MiBa among 3,620 total tests. Overall, 32 duplicate observations in the dataset were excluded from analysis. The number of non-POC tests that had a correspondent POC test was 27 (0.1%) in season 2016/17, 1,999 (3%) in season 2017/18 and 995 (2%) in season 2018/19. Overall, the percentage of positive influenza tests per season did not decrease after the introduction of POC testing: it was 17%, 22% and 17% respectively in seasons 2014/15, 2015/16 and 2016/17, while it was 27% and 20% in seasons 2017/18 and 2018/19. Rather, the mean seasonal percentage of positive tests (measured at regional level) was higher after the introduction of POC tests (21% ± 0.5 SD before vs 26% ± 0.5 SD after; p value < 0.05). The percentage of positive tests varied among regions and within a season, with the highest percentages observed in North Denmark Region and Region Zealand (in all seasons). The highest percentage of positive tests was seen in the 5–18-year-old group and the lowest in the 0–4-year-olds (Table 1). The countrywide seasonal influenza test incidence was higher after the introduction of POC testing in all age groups (Figure 2).

### Clinical Microbiology Departments questionnaire aggregated data

**Figure 3:**
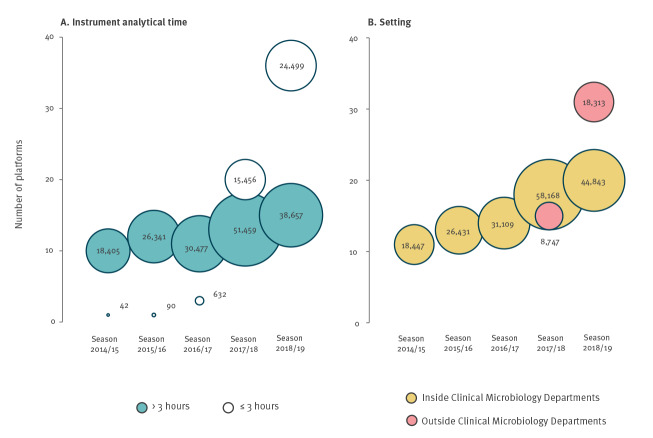
Seasonal number of influenza tests (circles) and number of operating platforms (vertical axis), Denmark, 2014–2019 (n = 206,058)

Source: Clinical Microbiology Departments.

From season 2017/18 to season 2018/19, there was an increase in (i) the seasonal number of tests performed, (ii) the number of tests performed with an instrument analytical time ≤ 3 h and outside a CMD and (iii) the number of operating platforms. The number of operating platforms with an instrument analytical time ≤ 3 h increased from 20 in season 2017/18 to 36 in season 2018/19 and the number of locations testing outside a CMD increased from 15 in season 2017/18 to 31 in season 2018/19 (Figure 3).

### Comparison of information on point-of-care tests in the Danish Microbiology Database with information from the Clinical Microbiology Departments questionnaire aggregated data

Table 2 shows an overall similarity between the numbers of tests registered as POC in MiBa and the number of tests performed with an instrument analytical time ≤ 3 h or outside a CMD (CMD questionnaire aggregated data) in the Capital Region of Denmark. On the other hand, there were exceptions: in season 2017/18, MiBa data showed 1,330 POC tests in Central Denmark Region, while 646 tests were conducted outside the CMD and 2,158 tests conducted within ≤ 3 h; similarly, the Region of Southern Denmark reported fewer tests conducted outside a CMD than tests reported as POC in MiBa.

**Table 2:** Number of influenza tests by source and type of test, by region, Denmark, 2017–2019

Region	Source	Type of test	Season 2017/18	Season 2018/19
Capital Region of Denmark	MiBa	Non-POC	27,229	20,604
		POC	6,331	8,939
	CMD	Inside CMD	26,986	21,695
		Outside CMD	6,420	8,998
		> 3h IAT	26,986	21,695
		≤ 3h IAT	6,420	8,998
North Denmark Region	MiBa	Non-POC	3,247	2,295
		POC	0	0
	CMD	Inside CMD	3,166	2,288
		Outside CMD	0	0
		> 3h IAT	3,166	2,288
		≤ 3h IAT	0	0
Central Denmark Region	MiBa	Non-POC	7,158	4,634
		POC	1,330	3,536
	CMD	Inside CMD	8,089	5,315
		Outside CMD	646	3,513
		> 3h IAT	6,577	4,020
		≤ 3h IAT	2,158	4,808
Region of Southern Denmark	MiBa	Non-POC	8,907	5,052
		POC	5,954	8,982
	CMD	Inside CMD	13,457	9,434
		Outside CMD	1,681	4,983
		> 3h IAT	8,260	4,543
		≤ 3h IAT	6,878	9,874
Region Zealand	MiBa	Non-POC	6,332	6,817
		POC	0	0
	CMD	Inside CMD	6,470	6,111
		Outside CMD	0	819
		> 3h IAT	6,470	6,111
		≤ 3h IAT	0	819

CMD: Clinical Microbiology Department (n = 130,071); IAT: instrument analytical time; MiBa: Danish Microbiology Database (n = 127,347); POC: point-of-care.

**Figure 4:**
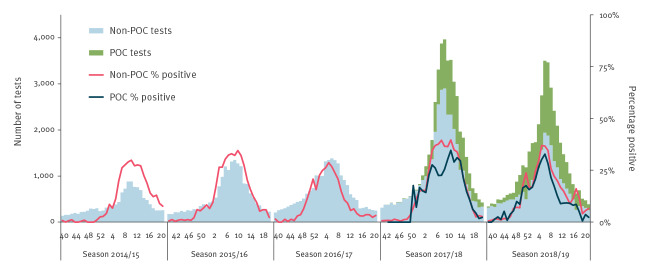
Seasonal weekly number of influenza tests (non-point-of-care/point-of-care) and percentage positive in CMD that show a correspondence in data from the Danish Microbiology Database and the CMD, Denmark, 2014–2019 (n = 141,644)

In five CMD, the number of POC tests registered in MiBa and the number of tests with an instrument analytical time ≤ 3 h differed by less than 5%. They represented 141,644 tests over the five seasons (71% of the overall number of performed tests in the study period). Figure 4 shows the weekly number of POC and non-POC influenza tests in MiBa and their percentage positive in these five CMD. In season 2017/18, the increasing number of performed tests was mainly represented by non-POC tests, while in season 2018/19 the relative proportion of POC tests increased. In both seasons, the percentage of positive POC tests resulted slightly lower than for non-POC tests (Figure 4).

CMD: Clinical Microbiology Departments.

## Discussion

To the best of our knowledge, this was the first study to explore the effect of POC testing on influenza surveillance from a nationwide perspective. The unique Danish setting, where a national surveillance system of all performed influenza tests is captured in a single database, allowed us to explore the introduction of POC testing from the perspective of influenza surveillance [10]. After influenza POC testing was introduced countrywide in 2017, we observed an increase in the number of performed tests in both season 2017/18 and 2018/19. Overall, this increase was not followed by a drop in the percentage of positive influenza tests, which suggests that the indication and practice of performing a test did not change despite the availability of POC testing. However, the observed increase in the seasonal percentage of positive tests at the regional level has to be taken with caution, as other factors outside of the scope of this analysis may explain these findings, e.g. the different severity of influenza seasons. In addition, the difference in the percentage of positive influenza tests among regions and age groups did not change remarkably during the study period. During the two POC seasons, the percentage of positive influenza tests resulted slightly lower for POC compared with non-POC testing. We believe that these findings could not reasonably be affected by the one CMD reporting 207 POC tests in MiBa during season 2016/17. Finally, we identified a number of non-POC tests that had a correspondent POC test (same unique identifier, date of sampling, reporting CMD) starting in season 2016/17: these were likely to be POC tests conducted in the emergency room and then repeated (as non-POC tests) upon admission to the ward. Despite the inefficiency of such a case-management practice, we included these tests in our analysis to complete the picture of influenza surveillance before and after the introduction of POC testing. 

In Europe, influenza season 2017/18 lasted longer than usual, mainly driven by influenza type B. An increased severity of cases was observed, with 21 European countries reporting excess mortality. Similarly, season 2018/19 also had increased levels of severity (with 22 countries reporting excess mortality) but primarily due to influenza type A [18,19]. The severity of seasons 2017/18 and 2018/19 – reflected in Denmark in a rise of influenza-related hospitalisations during those seasons – can partially explain the increase in the number of performed influenza tests and the number of positive influenza tests. Nonetheless, we cannot rule out that the availability of a rapid test method near the patients has resulted in more frequent testing compared with seasons where all influenza tests were performed only at the CMD [14,15].

Seasons 2017/18 and 2018/19 had more cases than the previous ones, but we found no conclusive evidence to ascribe the recent larger seasonal waves to POC testing (based on microbiological testing). Nonetheless, the number of available POC operating platforms also increased from one season to the next after its introduction and this could explain the relative larger share of POC tests in season 2018/19 compared with 2017/18. Finally, we also observed variations in the operational implementation of POC testing, with CMD reporting proportionally more tests conducted near to the patient than others.

Denmark implemented local and regional POC committees collaborating with CMD to assess the needs for testing assays of clinical relevance, defining the placement of instruments and their operational procedures, identifying and training the personnel responsible for operating POC testing, and ensuring communication and quality control practices. We want to emphasise the importance of an integrated collaboration among CMD, local POC committees and the Danish Society for Clinical Microbiology. These organisations work together to improve the quality of testing and make sure that also near-patient tests are registered in the national MiBa database [13].

The primary goal of implementing POC testing is the clinical management of patients, from diagnosis to treatment and follow-up [4,20]. Different clinical settings pursue different goals, e.g. an emergency room (when the admission of the patient may be driven by infection control purposes such as isolation at admission) vs a department of internal medicine (deciding about starting an antiviral therapy) [21-23]. Having testing platforms available outside the CMD and with a shorter instrument analytical time is intuitively suggestive of more testing but this did not seem to affect the relevance and outcome of influenza tests in Denmark. Given the unique Danish setting and these encouraging results (i.e. no drop in the percentage of positive tests), we propose the utilisation of POC results in the routine surveillance of seasonal influenza. A few authors have already suggested that seasonal response can improve with real-time monitoring via POC testing [24-26]. Also, de Lusignan et al. inquired if POC testing could have a place at the level of general practices for influenza-like illness and influenza surveillance purposes [27]. The experience with POC testing in Denmark reminds us how fast diagnostic procedures, patient care and the related reporting of data are changing and are more interconnected. While real-time data has become fundamental for disease surveillance – not only of influenza – we see the importance of standardised approaches, e.g. to harmonise testing and reporting procedures, and machines at the peripheral level [13,28]. The Danish setup, with only 11 CMD distributed in five regions, offers operational and logistical advantages that should not be overlooked.

We believe that the Danish nationwide experience in implementing POC testing for influenza can be relevant to better understand the challenges of disease surveillance at country level in Europe, before and after the implementation of POC testing.

The main limitation of this study was its empirical approach to link surveillance data from MiBa with data from the CMD and to assess their correspondence. This was due to the absence of an operational definition for an influenza POC test. We categorised tests as performed inside/outside a CMD and with ≤/> 3 h analytical time, but this may not fully account for the role played by distances between clinical settings and CMD and by the time lag between the moment a test is ordered, the sample is taken and the result is available for clinical purposes. In addition, data on whether a test was classified as POC (yes/no) in MiBa had to be retrieved from information about the analysis performed or from a commentary field, indicating that the reporting of POC tests in MiBa was not yet fully standardised when this study was conducted. Nor did our study explore the role of other factors that may be associated with the disease burden and its severity on a seasonal basis. However, these aspects were deemed outside of the scope of a study that aimed at describing the possible changes occurring in influenza surveillance after the introduction of POC testing in Denmark. Finally, we should mention additional factors that may have played a role but are difficult to assess, such as the relative novelty of POC procedures to the staff implementing it, the fast increasing number of instruments at facility level from 2017/18 to 2018/19 and the assumed increase in the number of staff operating them (with potentially diverse professional backgrounds). The short study period, including only three and two influenza seasons before and after the introduction of POC testing which were different in their magnitude and circulating strains, is another factor that might have affected our findings.

### Conclusion

We were able to describe influenza trends before and after the introduction of POC testing in Denmark, and none of our findings suggested any evident effect on influenza surveillance. The availability of POC testing did not result in a decrease in the percentage of positive influenza tests, despite an increased number of tests in both season 2017/18 and 2018/19. This study highlights the importance of capturing POC test results into the national laboratory system, which remains a strength of the Danish setting. Also, it supports evidence that POC testing may play a substantial, active role in influenza surveillance. However, further efforts are necessary to align testing procedures and machines among facilities and CMD and to ensure the full standardisation of POC test reporting.
